# Safety of orogastric tubes in foregut and bariatric surgery

**DOI:** 10.1007/s00464-018-6269-y

**Published:** 2018-06-19

**Authors:** Kulvir Nandra, Richard Ing

**Affiliations:** Department of Surgery, Thomas Jefferson University Hospital, 1015 Walnut Street, Curtis Building, Suite 620, Philadelphia, PA 19107 USA

**Keywords:** Orogastric tube complications, Bougie, GastriSail, ViSiGi 3D, Sleeve gastrectomy

## Abstract

**Background:**

Orogastric tubes have traditionally aided foregut procedures with sizing and organ protection. The rise of bariatric surgery has led to the creation of novel medical devices aimed at facilitating the laparoscopic sleeve gastrectomy. While approved by the FDA, the long-term safety profile of these devices in the general population is often unknown. This review looks at complications associated with novel Orogastric Tubes compared to the traditional bougie.

**Methods:**

We performed a review of the Food and Drug Administration’s (FDA) Manufacturer and User Facility Device Experience (MAUDE) database for complications associated with the traditional bougie, Boehringer Labs ViSiGi 3D® and the Medtronic GastriSail™ since 2011. In addition, we looked for reported cases in the literature of complications with these devices.

**Results:**

Overall complication rates reported in the MAUDE database varied in number and severity. The bougie had seven reported complications, one of which was an organ perforation. The ViSiGi 3D® had zero reported complications. The GastriSail™ had 36 total reported complications with 17 perforations. A literature review shows that rates of bougie complications are extremely rare with no case reports or reviews of complications from the novel orogastric tubes.

**Conclusions:**

The complication rates between the traditional bougie and novel devices vary in number and severity, with the GastriSail™ having the highest reported complication rate. Despite rigorous testing for FDA approval, ongoing research into performance of new medical devices in the general population remains important.

Since the advent of foregut and bariatric surgery, intraluminal devices have been used to identify position and guide key steps of these procedures. These devices prevent narrowing and act as sizers for the creation of new structures. Both weighted and unweighted bougies have traditionally been used to dilate and measure in esophageal and gastric procedures. The recent popularity of vertical sleeve gastrectomy has driven innovation into devices that can create a uniform and untwisted gastric pouch without stenosis. While there remains no consensus in gastric sleeve sizing, with bougie size ranging from 32 to 50 F, there is agreement that a lesser curve-based pouch is optimal [1]. Traditional bougies can be difficult to maintain in proper position intra-operatively [2]. Recently, two devices have become available for usage: Boehringer Labs ViSiGi 3D® and the Medtronic GastriSail™. This review evaluates the risk profile of these two devices as compared to usage of a traditional bougie.

## Methods

A thorough review of the literature was performed as well as an evaluation of the Manufacturer and User Facility Device Experience (MAUDE) database kept by the Food and Drug Administration. This database catalogues all self-reported adverse events from all medical devices [3].

## Results

Since 2011, bougies have seven total device malfunctions reported to the FDA. As an older device, several companies make bougies today. Five device spills occurred with use of the Teleflex brand bougie which contains internal mercury that can leak during suturing. These spills led to no changes in patients’ clinical statuses. A Medovations lighted bougie had a breakage at a lighted tip with retained foreign body in patient’s oropharynx which led to temporary reintubation. The GastriSail™ has 35 reported complications since introduction in 2015. This includes 17 perforations of either the stomach or esophagus and 11 device malfunctions. Lastly, the ViSiGi 3D® has no reported complications since its usage began in 2014. The breakdown of complications by device is displayed on Table 1. GastriSail™-related complications required foreign body retrieval for 7 cases with all 17 perforations reportedly leading to organ repair or esophageal stent placement. In total, 24 (68%) of GastriSail™ complications needed subsequent intervention. Bougie complications led to one foreign body retrieval and one perforation repair with 2/7 (28%) of complications requiring intervention (Table 2). Lastly, a literature review revealed no published cases or reviews of complication rates from either the ViSiGi 3D® or GastriSail™. Bougie-related complications are rarely described, and a handful of case reports and retrospective studies related to are published [4, 5].

**Table 1:** MAUDE review

Complication	Bougie	GastriSail™	ViSiGi 3D®
Device spill	5	0	0
Perforation	1	17	0
Device entrapment	0	5	0
Miscellaneous injury	0	2	0
Malfunction	0	11	0
Retained foreign body	1	1	0
Total	7	36	0

**Table 2:** Interventions

Intervention	Bougie	GastriSail™	ViSiGi 3D®
Foreign body retrieval	1	7	0
Repair of perforation	1	17	0
Total	2 (28%)	24 (68%)	0

## Discussion

With a long history of aiding foregut surgery, the safety profile of bougies can be adequately estimated from the literature and MAUDE database. A recent review by Zhang et al. found only two cases of esophageal perforations related to bougie insertion out of 1223 reviewed foregut surgery cases [5]. All cases of perforation require further intervention such as esophageal stenting, primary repair, diversion, or even esophagectomy in severe cases [6]. Individual case reports highlight the morbidity of iatrogenic esophageal perforation which can have a delayed presentation and lead to death even after intervention [7]. Bougie-related injuries can occur from improper traction or anesthesia inexperience, suggesting that bougie placement is best handled by trained members of an operating team [5]. Nevertheless, rates of bougie complications, especially perforations, are extremely rare. Design modifications over time, such as softer tubes, and experienced bougie insertions may have contributed to a reduction in these complications [5]. The current literature offers no safety profiles of the novel orogastric tubes currently in use.

It should be noted that MAUDE reports cannot accurately determine incidence of complications due to underreporting and lack of verification of reports [3]. It is difficult, then, to make conclusions based off a MAUDE analysis alone. Nevertheless, a review of these reports shows a clear disparity in the safety of different orogastric tube devices, with the GastriSail™ having a higher reported incidence of complications. Thus, we can demonstrate variability in the safety profile of novel orogastric tube devices used in the laparoscopic sleeve gastrectomy. While it is challenging to assess the true complication rate, surgeons must be vigilant in monitoring poor outcomes from these devices and ensure all staff receives adequate training. Even with a rigorous approval process from the FDA, the long-term safety of new devices among a diverse population of surgeons must be researched.
